# Identification and Analysis of Key lncRNAs for Adipose Differentiation

**DOI:** 10.3390/biology15010087

**Published:** 2025-12-31

**Authors:** Xiujie Xie, Tianyu Li, Bohang Zhang, Junxiong Liao, Xing Zhang, Jing Gao, Xiaofang Cheng, Tiantian Meng, Yongjie Xu, Pengpeng Zhang, Cencen Li

**Affiliations:** College of Life Sciences, Xinyang Normal University, Xinyang 464000, Chinameng2023@xynu.edu.cn (T.M.);

**Keywords:** lncRNAs, adipose tissue, WAT browning

## Abstract

Recent studies have demonstrated that the abundance of brown adipose tissue is inversely associated with obesity in humans. Promoting the browning of white adipocytes therefore represents a promising therapeutic strategy for obesity treatment. LncRNAs are known regulators of adipocyte differentiation and metabolic processes. However, their specific roles in adipocyte browning remain poorly characterized. In this study, we performed transcriptomic analyses using publicly available RNA-seq datasets of mouse white, brown and beige adipose tissues from the EMBL-EBI database. Our analytical workflow included raw data quality control, alignment to the reference genome, transcript assembly, coding potential assessment and differential expression analysis. Functional annotation was conducted through Gene Ontology (GO) and Kyoto Encyclopedia of Genes and Genomes (KEGG) enrichment analyses. Key lncRNAs were further validated via Reverse Transcription Quantitative PCR (RT-qPCR). We identified 794 novel lncRNAs and 1499 DEGs, among which 95 were common across all three adipocyte types. Two lncRNAs, *MSTRG.12661* and *MSTRG.17758*, were found to be closely related to critical biological processes, including extracellular matrix organization and fatty acid oxidation. Functional prediction suggests their potential involvement in adipocyte type-specific differentiation. In conclusion, our study reveals novel lncRNAs that may regulate adipocyte differentiation, offering new candidate targets for obesity treatment via induction of white adipose tissue browning.

## Simple Summary

Long non-coding RNAs (lncRNAs) are well-recognized regulators of adipocyte differentiation and metabolic processes. However, their specific roles in adipocyte browning remain poorly characterized. To address this knowledge gap, we performed transcriptomic analyses on publicly available RNA-seq datasets of mouse white (WAT), brown (BAT), and beige (BeAT) adipose tissues; these datasets were retrieved from the EMBL-EBI database under the accession number E-MTAB-2624. Through a sophisticated bioinformatics analysis pipeline, we ultimately identified 794 novel lncRNAs and 1499 differentially expressed genes (DEGs), among which 95 were shared across all three adipocyte types. Among these novel lncRNAs, two specific lncRNAs, *MSTRG.12661* and *MSTRG.17758*, were found to be tightly associated with key biological processes, including extracellular matrix organization and fatty acid oxidation; functional prediction further indicates their potential involvement in adipocyte type-specific differentiation. To conclude, our study identified novel lncRNAs potentially involved in regulating adipocyte differentiation, providing new candidate targets for treating obesity by inducing white adipose tissue browning.

## 1. Introduction

When dietary energy intake chronically exceeds the energy expended for growth and metabolism, surplus lipids, carbohydrates, and other nutrients are converted into triglycerides [1], which are stored as fat and contribute to the development of obesity [2]. Obesity is a major risk factor for numerous metabolic disorders, including type 2 diabetes and cardiovascular diseases, and has also been linked to an increased risk of several cancers [3,4,5,6,7]. It is estimated that over 4 million deaths annually are attributable to excess body weight [8]. Adipose tissue, composed primarily of lipid-storing adipocytes, serves as the body’s main energy reservoir and is classified into three major types: white, brown, and beige adipose tissue [9].

White adipose tissue is the most abundant form and is widely distributed in mammals. White adipocytes are characterized by a single large lipid droplet that displaces the nucleus to the periphery [10]. Beyond energy storage, WAT contributes to thermal insulation and endocrine regulation of systemic energy homeostasis [11]. In contrast, brown adipose tissue is thermogenically active, especially in neonates and under cold conditions [12,13]. Its brown coloration stems from high vascularity and mitochondrial density [14]. Brown adipocytes contain multilocular lipid droplets and express high levels of uncoupling protein 1 (*UCP1*), which dissipates the mitochondrial proton gradient to produce heat instead of ATP [15].

Beige adipose tissue arises within WAT depots in response to stimuli such as cold exposure, exercise, or pharmacological agents [16]. This process, termed the “browning”, leads to a cell type with thermogenic capacity similar to BAT [17]. Promoting WAT browning has emerged as a promising strategy to counter obesity and metabolic disease by enhancing energy expenditure.

LncRNAs are transcripts longer than 200 nucleotides with limited or no protein-coding potential, often exhibiting cell- or tissue-specific expression [18]. They participate in diverse biological processes-including epigenetic regulation, cell differentiation, and metabolic signaling-through various mechanisms of gene expression control [19].

Growing evidence implicates lncRNAs in the regulation of adipocyte differentiation. For instance, Sun identified 175 lncRNAs dynamically regulated during adipogenesis [20], while Wang revealed the involvement of *MEK6-AS1* in adipogenic processes [21]. Mostafa proposed *LINC00312*, *LINC00607*, and *TYMSOS* as key regulators in white adipogenesis [22]. Moreover, Tran reported that the primate-specific *LINC00473* is highly expressed in human thermogenic adipocytes, correlates with *UCP1*, and is reduced in obesity and type-2 diabetes [23].

To further elucidate the role of lncRNAs in adipose biology, we analyzed RNA-seq data from mouse WAT, BAT, and BeAT. Our objectives were to identify novel adipose-associated lncRNAs and refine existing annotations. Through differential expression analysis, we aimed to pinpoint key lncRNAs involved in adipocyte differentiation and browning. This study enhances the understanding of lncRNA-mediated regulatory mechanisms in adipose tissue and may contribute to identifying novel therapeutic targets for obesity and related metabolic disorders.

## 2. Materials and Methods

### 2.1. Animals and Tissue Preparation

All animal experiments in this study were approved by the Institutional Review Board of Xinyang Normal University. Mice were housed under conditions of 40–70% humidity and an ambient temperature of 22 ± 2 °C, with a standard 12 h light/12 h dark cycle (8:00 a.m.–8:00 p.m.), and had ad libitum access to food and water. The basal diet used in this study was purchased from Xietong (Nanjing, China). In the study, 6 healthy 8-week-old male C57BL/6 mice (body weight: ~20.0 g) were randomly allocated into two experimental groups, namely the control group and the cold stimulation group, with 3 mice per group serving as biological replicates (*n* = 3). Specifically, mice in the control group were reared at room temperature, while those in the cold stimulation group were maintained in a constant-temperature environment of 4 °C. Except for the temperature condition, all other rearing conditions were kept consistent between the two groups. The experimental treatment lasted for 3 days. After the treatment, all mice were euthanized by cervical dislocation. The mice were rapidly dissected to collect major organs, including the heart, spleen, lung, and kidney, as well as BAT and WAT. All collected samples were immediately transferred to a −80 °C ultra-low temperature refrigerator for storage until subsequent experimental analysis.

### 2.2. Quality Control Procedures of RNA-Seq Data

RNA-seq datasets corresponding to mouse white, beige, and brown adipocytes were retrieved from the EMBL-EBI database under accession E-MTAB-2624 [24]. The dataset included two biological replicates for each adipocyte type (Table 1). Raw sequence files in SRA format were converted to FASTQ format using the fastq-dump utility with default parameters. Quality assessment of the raw reads was performed using FastQC (v0.11.5) [25,26] to verify that all samples met standard quality criteria prior to downstream analysis.

### 2.3. RNA-Seq Data Analysis

Raw sequencing reads were initially processed with Trimmomatic (v0.33) [27] to remove adapter sequences and low-quality bases. The trimming steps included: (1) adapter removal; (2) Sliding window trimming (4-base window, minimum Phred quality score of 15); and (3) exclusion of reads shorter than 63 nucleotides after trimming.

The high-quality reads were then aligned to the mouse mm9 reference genome (genome.NCBIM37.clean.fa) using HISAT2 (v2.1.0) [28]. The resulting SAM files were sorted and converted to BAM format using SAMtools (v1.9) [29]. Transcript assembly was performed with StringTie (v2.0) [30], and individual transcriptomes from all samples were merged into a unified annotation file using the StringTie --merge function.

### 2.4. Identification of Novel lncRNAs

Novel lncRNAs were identified from the merged transcriptome annotation through a multi-step filtering pipeline implemented with custom Python (V2.7) scripts (File S1). Transcripts were retained if they met the following criteria: (1) length > 200 nucleotides and an FPKM > 0.5 in at least one sample; (2) no overlap with known exons and located >2 kb away from any single-exon transcript; and (3) coding potential score < 0 as predicted by both CPAT (V1.2.4) [31] and COME (V1.4.0) [32] tools.

### 2.5. Characterization of Novel lncRNAs

A systematic comparison was conducted among newly identified lncRNAs, annotated lncRNAs, and protein-coding genes based on their biophysical and expression features, including transcript length, expression level (FPKM), coding potential, and conservation. The results of these comparative analyses are presented using the ggplot2 (V 3.4.4) package in R (V 4.3.3).

### 2.6. Methods for Differential Expression Analysis and Functional Annotation

Differential expression analysis of both novel and annotated lncRNAs across the three adipose tissue types was conducted using the DESeq2 (V1.40.2) package in R. Significantly differentially expressed lncRNAs were defined as those with an adjusted *p*-value (*padj*) < 0.05 and an absolute log2 fold change >1. Putative target genes of the differentially expressed lncRNAs were predicted, and their potential biological functions were investigated through GO and KEGG pathway enrichment analyses to identify key lncRNAs implicated in adipocyte differentiation.

### 2.7. Total RNA Extraction and RT-qPCR

Total RNA of tissues was extracted and purified using RNAiso Plus (TaKaRa, Dalian, China) according to manufacturer’s instructions. Total RNA was dissolved in RNase-free H_2_O, and the concentration and purity were measured using a NanoDrop 2000 spectrophotometer (Thermo Scientific, Waltham, MA, USA). All samples had absorbance ratios of 1.8–2.0 at 260 and 280 nm. RNA integrity and contamination were verified by 1% agarose gel electrophoresis. 1 μg of total RNA from different samples was prepared using a reverse transcription kit (TaKaRa, Dalian, China) to be reverse-transcribed into cDNA. RT-qPCR reactions were performed using TB Green^®^ Fast qPCR Mix (TaKaRa, Dalian, China) and the CFX-96 Real-Time PCR Detection System (Bio-Rad, Hercules, CA, USA). Relative expression levels were calculated using 2^−ΔΔt^ method, and 18S rRNA was used as the internal reference gene for normalization. All primer sequences (Table 2) were synthesized by General Biol (Anhui) Co., Ltd. (Chuzhou, China).

## 3. Results

### 3.1. Quality Control Results of RNA-Seq Data

Initial quality assessment of the raw RNA-seq data confirmed its high quality and suitability for downstream analysis. Key metrics included an N-content of 0 across all sequencing positions, an average base quality score peaking at 38, and per-base quality scores consistently above 28. The observed GC content distribution across sequences aligned well with the theoretical distribution, indicating no significant bias or contamination (Figure S1).

### 3.2. Identification and Characterization of lncRNAs

**Figure 1:**
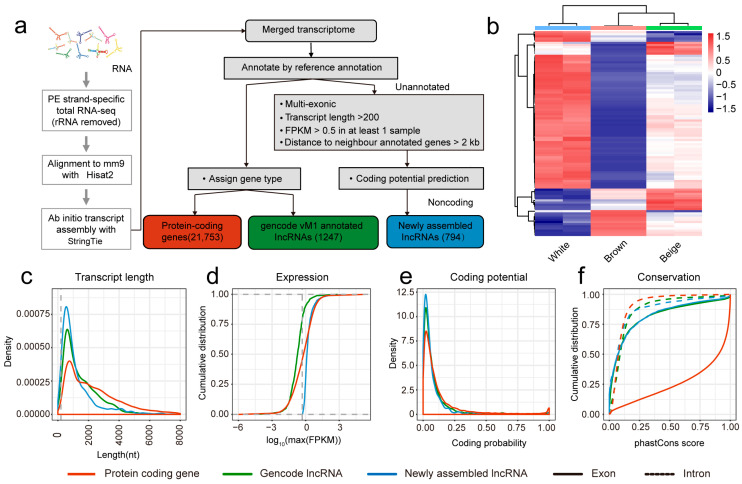
Identification of newly assembled lncRNAs: (**a**) LncRNA identification flowchart; (**b**) Heatmap of differentially expressed newly assembled lncRNAs; (**c**) Density curves for transcript length; (**d**) Cumulative distribution curves of gene expression; (**e**) Density curves of gene-encoded potential; (**f**) Cumulative distribution curves of gene conservatism.

Transcriptomic alignment and assembly identified 21,753 annotated protein-coding genes and 1247 known lncRNAs. A stringent filtering pipeline was applied to the remaining unannotated transcripts to identify novel lncRNAs (Figure 1a). This multi-step process, based on exon count, transcript length (>200 bp), expression level (FPKM > 0.5 in at least one sample), and coding potential, yielded 794 novel lncRNAs (Files S2–S5). A global view of gene expression in WAT versus BAT showed that the majority of genes were expressed at higher levels in WAT (Figure 1b).

Comparative analysis revealed distinct features of the novel lncRNAs. Both novel and annotated lncRNAs were typically shorter than protein-coding genes, which showed a greater proportion of transcripts exceeding 2 kb (Figure 1c). As expected, lncRNAs exhibited lower expression levels than protein-coding genes. Notably, the novel lncRNAs were expressed at a higher level than annotated lncRNAs (Figure 1d). Coding potential analysis confirmed that the novel lncRNAs had minimal protein-coding capacity, with scores significantly lower than those of annotated lncRNAs and protein-coding genes (Figure 1e). Conservation analysis indicated that exon regions of both novel and annotated lncRNAs were less conserved than those of protein-coding genes, while all three categories showed low conservation in intronic regions (Figure 1f).

### 3.3. Differential Expression Analysis and Functional Annotation

**Figure 2:**
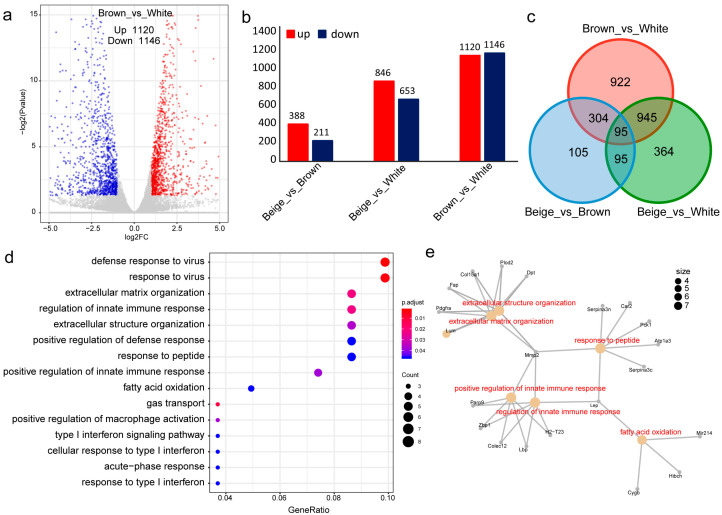
Differential expression analysis of lncRNAs: (**a**) Volcano plot of differential lncRNAs; (**b**) Histogram of data comparison of differential lncRNAs up-and-down in white, brown and beige adipocytes; (**c**) Venn Diagram of overlapping lncRNA data in white, brown, and beige adipocytes; (**d**) GO enrichment analysis bubble plot of differential lncRNAs; (**e**) Gene network diagram of differential lncRNAs.

Differential expression analysis between BAT and WAT identified 2266 differentially expressed genes, with 1120 upregulated and 1146 downregulated in WAT relative to BAT. Among these, 181 lncRNAs exhibited differential expression between BAT and WAT (Figure 2a,b). Comparison of BeAT and WAT revealed 1499 DEGs, with 846 upregulated and 653 downregulated in WAT. Specifically, 74 and 31 lncRNAs were upregulated and downregulated in WAT, respectively (Figure 2b). The contrast between BeAT and BAT yielded 599 DEGs, including 45 differentially expressed lncRNAs (Figure 2b). An intersection analysis identified 95 common DEGs consistently differentially expressed across all three adipose cell types (Figure 2c).

GO enrichment analysis of these 95 common DEGs highlighted significant involvement in biological processes critical to adipose biology, including extracellular matrix organization and fatty acid oxidation (Figure 2d). This suggested their potential regulatory roles in adipocyte differentiation. Notably, two lncRNAs, *MSTRG.12661* and *MSTRG.17758*, were prioritized as key candidates. A gene network analysis further implicated two genes (*Cygb* and *Hibch*) and one microRNA (*mir214*) as potential players in fatty acid oxidation pathways (Figure 2e).

### 3.4. Functional Prediction of Candidate lncRNA

**Figure 3:**
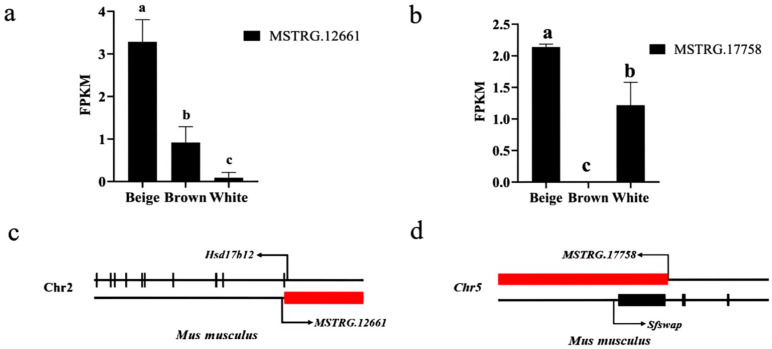
Expression and target genes of candidate lncRNA: (**a**) The FPKM value of *MSTRG.12661*; (**b**) The FPKM value of *MSTRG.17758*; (**c**) Positional relationship between *MSTRG.12661* and *Hsd17b12*; (**d**) Positional relationship between *MSTRG.17758* and *Sfswap*. The different letters (a, b, c) above the bars in (**a**) and (**b**) revealed that the FPKM expression levels of MSTRG.12661 and MSTRG.17758 were significantly different among groups (*p* < 0.05).

Expression profiling showed that *MSTRG.12661* was highly expressed in beige adipocytes but expressed at low levels in white adipocytes (Figure 3a). Genomic localization analysis placed *MSTRG.12661* on the antisense strand downstream of the *Hsd17b12* gene (Figure 3c), a known regulator of adipocyte differentiation. This positional relationship suggests a potential role for *MSTRG.12661* in modulating adipocyte differentiation.

The second candidate, *MSTRG.17758*, was also highly expressed in beige fat and absent in brown adipocytes (Figure 3b). This lncRNA was located upstream of *Sfswap* (Figure 3d), a protein-coding gene involved in mRNA splicing. We hypothesize that *MSTRG.17758* may influence adipocyte differentiation by interacting with *Sfswap* to regulate the splicing of key transcripts.

### 3.5. Validation of Gene Expression in RNA-Seq

**Figure 4:**
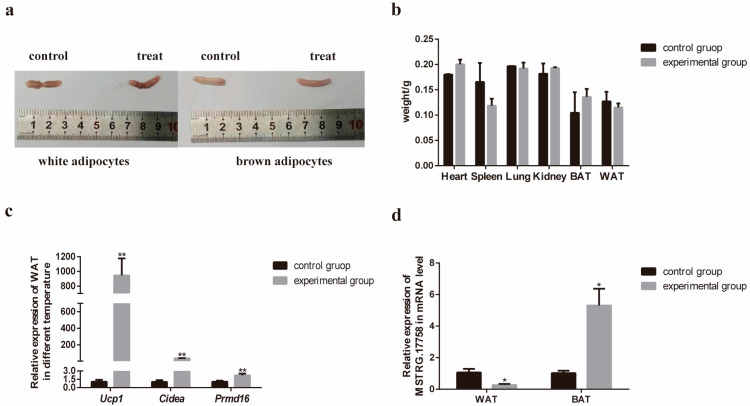
Validation of gene expression in RNA-seq under cold stimulation conditions: (**a**) Images of two groups of white adipose tissue and brown adipose tissue after treatment; (**b**) The weight of each tissue in the two groups after treatment (*n* = 3); (**c**) Relative expression levels of *Ucp1*, *Cidea* and *Prdm16* in WAT under room temperature and cold stimulation conditions (*n* = 3); (**d**) Relative expression levels of *MSTRG.17758* in WAT and BAT under room temperature and cold stimulation conditions (*n* = 3). Data are presented as the mean ± SEM from three biological replicates. * *p* < 0.05 and ** *p* < 0.01.

To investigate the regulatory effects of cold stimulation on mouse tissues, we compared tissue morphological differences between the cold-stimulated group and the room temperature-cultured group, with the cold stimulation treatment lasting for 3 consecutive days. There was no significant difference in the morphology of the adipose tissue between the two groups (Figure 4a). Subsequently, we collected the heart, spleen, lung, kidney, brown adipose tissue (BAT), and white adipose tissue (WAT) from both the control and experimental groups for weighing. The results indicated no significant differences in body weight between the two groups (Figure 4b). To further evaluate the reliability of the RNA sequencing data, we first examined the relative expression levels of three adipose tissue-specific marker genes (*Ucp1*, *Cidea*, and *Prdm16*) in WAT following cold stimulation via RT-qPCR. Our data revealed a statistically significant reduction in the expression of these genes in the experimental group versus the control group. (Figure 4c). Meanwhile, we also detected the expression levels of these three marker genes in BAT, and their relative expression levels were significantly increased compared with the control group; this result is presented in Figure S2. Additionally, a candidate gene (*MSTRG.17758*) was randomly selected for RT-qPCR validation. The results demonstrated that the expression of this gene was significantly decreased in white adipose tissue but significantly increased in brown adipose tissue, a trend fully consistent with the RNA-seq data (Figure 4d).

Collectively, these findings indicate that the RNA-seq data obtained in this study are highly reliable and accurate, providing a solid foundation for further investigation into the mechanisms by which cold stimulation regulates adipose metabolism in mice.

## 4. Discussion

In this study, we employed a comprehensive transcriptomic analysis of mouse white, beige, and brown adipose tissues to elucidate the landscape of long non-coding RNAs. Our investigation led to the identification of 794 novel lncRNAs, significantly expanding the catalog of non-coding transcripts associated with adipocyte biology. The molecular characterization of these transcripts reinforced the established paradigm that lncRNAs generally exhibit shorter lengths, lower expression, lower coding potential, and reduced evolutionary conservation compared to protein-coding genes [33]. This consistency with canonical lncRNA features underscores the robustness of our identification pipeline and provides a reliable foundation for further functional exploration.

Beyond cataloging, our comparative analysis across the three adipocyte types revealed distinct lncRNA expression signatures, suggesting their specific roles in defining adipose tissue identity and function. Notably, we pinpointed two lncRNAs, *MSTRG.12661* and *MSTRG.17758*, as high-priority candidates involved in the browning of white adipose tissue. The genomic context of *MSTRG.12661*, located downstream of the *Hsd17b12* gene, is particularly intriguing. *Hsd17b12* encodes 17β-hydroxysteroid dehydrogenase type 12, a key enzyme in fatty acid elongation and lipid metabolism [34]. The antisense orientation and proximity of *MSTRG.12661* to this metabolic regulator suggest a potential cis-regulatory mechanism, possibly influencing *Hsd17b12* expression to modulate lipid utilization and energy dissipation, thereby promoting a beige adipocyte phenotype.

Similarly, *MSTRG.17758*, which is upregulated in beige fat and located upstream of the *Sfswap* gene, presents another compelling case. *Sfswap* encodes a splicing factor involved in alternative mRNA splicing, a critical layer of post-transcriptional regulation [35]. It is plausible that *MSTRG.17758* interacts with *Sfswap* to influence the splicing patterns of a network of genes governing adipocyte differentiation and thermogenic programming. Such a mechanism would represent a novel pathway through which lncRNAs can orchestrate complex phenotypic changes, like the white-to-beige transition, by rewiring the transcriptome via splicing regulation.

While our bioinformatic predictions provide strong mechanistic hypotheses, they await experimental validation. A critical next step involves functional studies, such as gain-of-function and loss-of-function experiments in adipocyte cell models, to confirm the roles of *MSTRG.12661* and *MSTRG.17758* in browning. Furthermore, techniques like RNA antisense purification (RAP) or chromatin isolation by RNA purification (ChIRP) could be employed to verify the physical interactions between these lncRNAs and their putative genomic targets (*Hsd17b12*) or protein partners (*Sfswap*) [36,37]. Understanding the upstream regulators, such as specific transcription factors induced by cold exposure or β-adrenergic signaling [38], that control the expression of these lncRNAs would add another crucial layer to the regulatory network.

## 5. Conclusions

In conclusion, our study not only provides a valuable resource of novel adipocyte-associated lncRNAs but also delineates specific candidates with high potential for regulating adipose tissue browning. The proposed mechanisms—linking one lncRNA to lipid metabolism and another to mRNA splicing—highlight the diverse ways lncRNAs can fine-tune cellular identity. Future work focused on validating these pathways will not only advance our fundamental understanding of adipocyte plasticity but may also uncover novel, RNA-based therapeutic targets for combating obesity and its associated metabolic disorders.

## Figures and Tables

**Table 1:** Details of RNA-seq data.

Cell	Samples	Accession Number	Clean Reads	Mapped Reads	Mapping Rate
beige adipocyte	Beige-1	ERR525591	35,351,268	32,484,280	91.89%
beige adipocyte	Beige-2	ERR525593	64,167,168	59,944,968	93.42%
Brown adipocyte	Brown-1	ERR525592	41,512,791	38,540,475	92.84%
brown adipocyte	Brown2	ERR525589	43,076,724	40,759,196	94.62%
white adipocyte	White-1	ERR525590	42,978,995	39,020,629	90.79%
white adipocyte	White-2	ERR525594	32,916,998	30,333,013	92.15%

**Table 2:** Primer information table.

Gene	Primer Sequence
*Ucp1*	F: ATCTGGGCTTAACGGGTCCTCC R: TGCGAACCTCATCACTCGT
*Cidea*	F: CCTACGACATCCGATGCACA R: TATCCACGCAGTTCCCACAC
*Prdm16*	F: CCACAAGTCCTACACGCAGT R: GAGGGAGGAGGTAGTGCTGA
*18S*	F: ACCGCAGCTAGGAATAATGGA R: GCCTCAGTTCCGAAAACCA
*MSTRG.17758*	F: CTCGGTATGCACAATGCCAC R: CCTTATCACCTCCTGATGCCA

## Data Availability

We performed transcriptomic analyses using publicly available RNA-seq datasets of mouse white, beige, and brown adipocytes from the EMBL-EBI database (accession: E-MTAB-2624; originally from Shinoda et al., *Nat. Med.*, 2015 [24]). The newly generated data and information in this article are provided in the Supplementary Materials (Files S1–S5).

## Supplementary Materials

The following supporting information can be downloaded at https://www.mdpi.com/article/10.3390/biology15010087/s1, File S1: The analytical scripts used for data processing and analysis in the study; File S2: The FPKM values of genes across all samples; File S3: The count values of genes across all samples; File S4: Information of newly identified long non-coding RNAs in the study; File S5: The statistical information of differentially expressed genes; Figure S1: RNA-seq data quality control; Figure S2: Relative expression levels of *Ucp1*, *Cidea* and *Prdm16* in BAT under room temperature and cold stimulation conditions.
