# Supplementary material for: Identification and Analysis of Key lncRNAs for Adipose Differentiation
Source: Biology (Basel). 2025 Dec 31;15(1):87. doi: 10.3390/biology15010087 (PMC12785126; doi:10.3390/biology15010087)
Supplement: Supplementary file 1 [file biology-15-00087-s001.zip › 6. Figure S1.pdf]

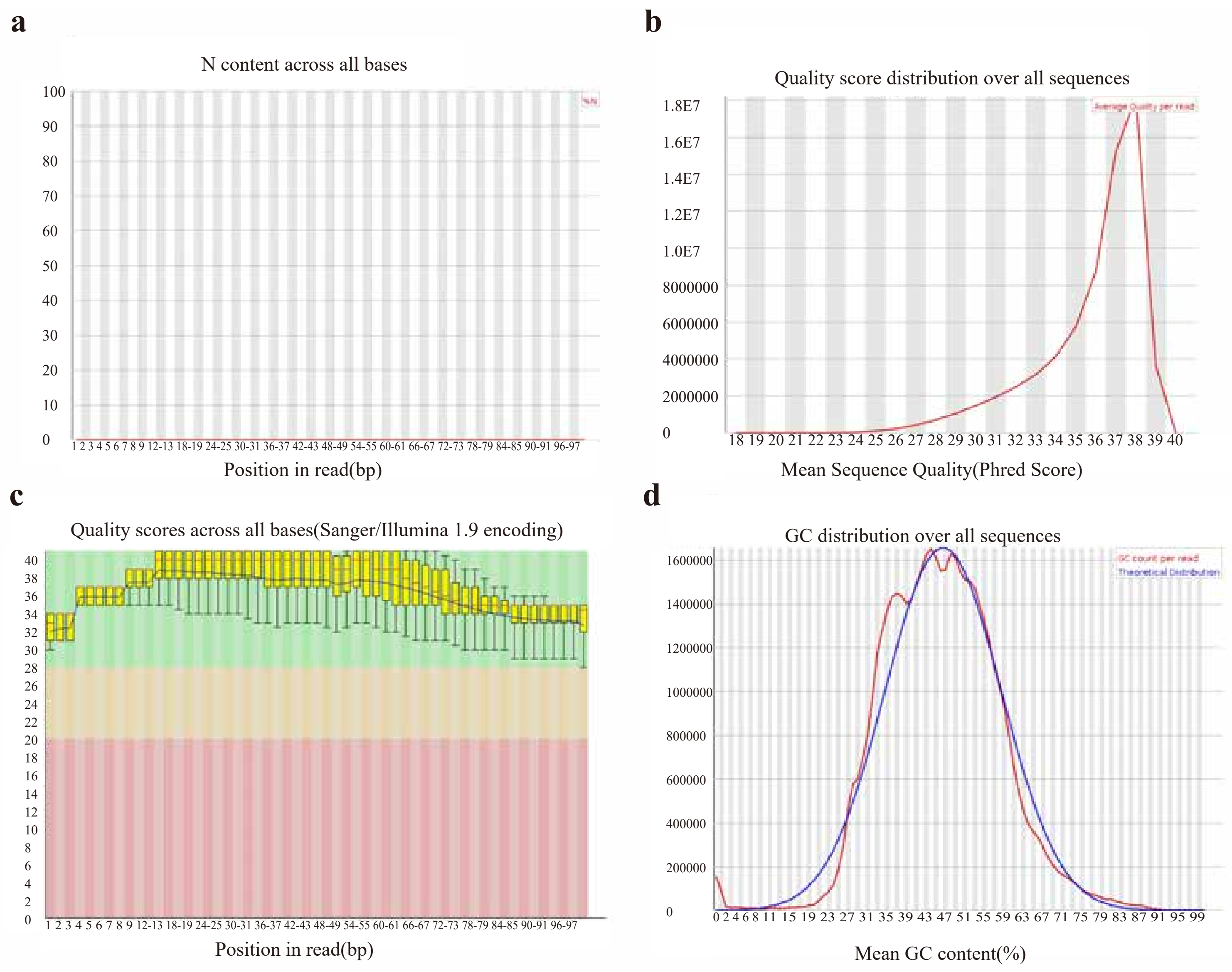

Figure S1. RNA-seq data quality control: (a) N content at each locus; (b) The average mass fre-quency of the sequence; (c) Base mass distribution corresponding to each site of the sequence; (d) Count the frequency of GC content for each sequence.
