# Supplementary material for: Identification and Analysis of Key lncRNAs for Adipose Differentiation
Source: Biology (Basel). 2025 Dec 31;15(1):87. doi: 10.3390/biology15010087 (PMC12785126; doi:10.3390/biology15010087)
Supplement: Supplementary file 1 [file biology-15-00087-s001.zip › 7. Figure S2.pdf]

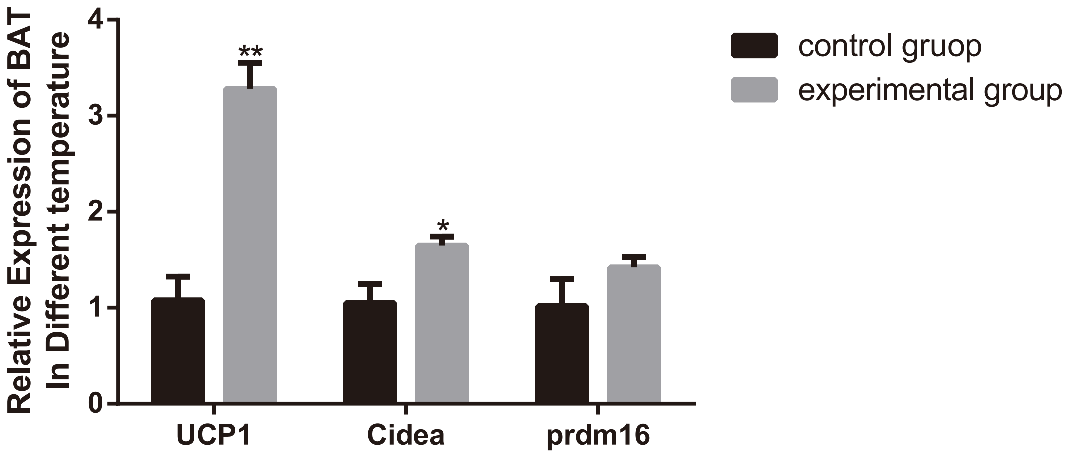

Figure S2. Relative expression levels of UCP1, Cidea and Prdm16 in BAT under room temperature and cold stimulation conditions (n = 3)
