# Supplementary material for: Identification and Analysis of Key lncRNAs for Adipose Differentiation
Source: Biology (Basel). 2025 Dec 31;15(1):87. doi: 10.3390/biology15010087 (PMC12785126; doi:10.3390/biology15010087)
Supplement: Supplementary file 1 [file biology-15-00087-s001.zip › supplementary material.pdf]

Supplementary Material

File S1. The analytical scripts used for data processing and analysis in the study.

File S2. The FPKM values of genes across all samples.

File S3. The count values of genes across all samples.

File S4. Information of newly identified long non-coding RNAs in the study.

File S5. The statistical information of differentially expressed genes.

File S5. The analytical scripts used for data processing and analysis in the study.

Figure S1. RNA-seq data quality control.

Figure S2. Relative expression levels of Ucp1, Cidea and Prdm16 in BAT under room temperature and cold stimulation conditions (n = 3).
